# Utilizing machine learning dimensionality reduction for risk stratification of chest pain patients in the emergency department

**DOI:** 10.1186/s12874-021-01265-2

**Published:** 2021-04-17

**Authors:** Nan Liu, Marcel Lucas Chee, Zhi Xiong Koh, Su Li Leow, Andrew Fu Wah Ho, Dagang Guo, Marcus Eng Hock Ong

**Affiliations:** 1 Duke-NUS Medical School, National University of Singapore, Singapore, Singapore; 2 Health Services Research Centre, Singapore Health Services, Singapore, Singapore; 3 Institute of Data Science, National University of Singapore, Singapore, Singapore; 4 Faculty of Medicine, Nursing and Health Sciences, Monash University, Melbourne, Australia; 5 Department of Emergency Medicine, Singapore General Hospital, Singapore, Singapore; 6 SingHealth Duke-NUS Emergency Medicine Academic Clinical Programme, Singapore, Singapore

**Keywords:** Machine learning, Dimensionality reduction, Heart rate n-variability (HRnV), Heart rate variability (HRV), Chest pain, Emergency department

## Abstract

**Background:**

Chest pain is among the most common presenting complaints in the emergency department (ED). Swift and accurate risk stratification of chest pain patients in the ED may improve patient outcomes and reduce unnecessary costs. Traditional logistic regression with stepwise variable selection has been used to build risk prediction models for ED chest pain patients. In this study, we aimed to investigate if machine learning dimensionality reduction methods can improve performance in deriving risk stratification models.

**Methods:**

A retrospective analysis was conducted on the data of patients > 20 years old who presented to the ED of Singapore General Hospital with chest pain between September 2010 and July 2015. Variables used included demographics, medical history, laboratory findings, heart rate variability (HRV), and heart rate n-variability (HRnV) parameters calculated from five to six-minute electrocardiograms (ECGs). The primary outcome was 30-day major adverse cardiac events (MACE), which included death, acute myocardial infarction, and revascularization within 30 days of ED presentation. We used eight machine learning dimensionality reduction methods and logistic regression to create different prediction models. We further excluded cardiac troponin from candidate variables and derived a separate set of models to evaluate the performance of models without using laboratory tests. Receiver operating characteristic (ROC) and calibration analysis was used to compare model performance.

**Results:**

Seven hundred ninety-five patients were included in the analysis, of which 247 (31%) met the primary outcome of 30-day MACE. Patients with MACE were older and more likely to be male. All eight dimensionality reduction methods achieved comparable performance with the traditional stepwise variable selection; The multidimensional scaling algorithm performed the best with an area under the curve of 0.901. All prediction models generated in this study outperformed several existing clinical scores in ROC analysis.

**Conclusions:**

Dimensionality reduction models showed marginal value in improving the prediction of 30-day MACE for ED chest pain patients. Moreover, they are black box models, making them difficult to explain and interpret in clinical practice.

## Background

Chest pain is among the most common chief complaints presenting to the emergency department (ED) [1–3]. The assessment of chest pain patients poses a diagnostic challenge in balancing risk and cost. Inadvertent discharge of acute coronary syndrome (ACS) patients is associated with higher mortality rates while inappropriate admission of patients with more benign conditions increases health service costs [4, 5]. Hence, the challenge lies in recognizing low-risk chest pain patients for safe and early discharge from the ED. There has been increasing focus on the development of risk stratification scores. Initially, risk scores such as the Thrombolysis in Myocardial Infarction (TIMI) score [6, 7] and the Global Registry of Acute Coronary Events (GRACE) score [8] were developed from post-ACS patients to estimate short-term mortality and recurrence of myocardial infarction. The History, Electrocardiogram (ECG), Age, Risk factors, and initial Troponin (HEART) score was subsequently designed for ED chest pain patients [9], which demonstrated superior performance in many comparative studies on the identification of low-risk chest pain patients [10–17]. Nonetheless, the HEART score has its disadvantages. Many potential factors can affect its diagnostic and prognostic accuracy, such as variation in patient populations, provider determination of low-risk heart score criteria, specific troponin reagent used, all of which contribute to clinical heterogeneity [18–21]. In addition, most risk scores still require variables that may not be available during the initial presentation of the patient to the ED such as troponin. There remains a need for a more efficient risk stratification tool.

We had previously developed a heart rate variability (HRV) prediction model using readily available variables at the ED, in an attempt to reduce both diagnostic time and subjective components [22]. HRV characterizes beat-to-beat variation using time, frequency domain, and nonlinear analysis [23] and has proven to be a good predictor of major adverse cardiac events (MACE) [22, 24, 25]. Most HRV-based scores were reported to be superior to TIMI and GRACE scores while achieving comparable performance with HEART score [17, 24, 26, 27]. Recently, we established a new representation of beat-to-beat variation in ECGs, the heart rate n-variability (HRnV) [28]. HRnV utilizes variation in sampling RR-intervals and overlapping RR-intervals to derive additional parameters from a single strip of ECG reading. As an extension to HRV, HRnV potentially supplements additional information about adverse cardiac events while reducing unwanted noise caused by abnormal heartbeats. Moreover, HRV is a special case of HRnV when *n* = 1. The HRnV prediction model, developed from multivariable stepwise logistic regression, outperformed the HEART, TIMI, and GRACE scores in predicting 30-day MACE [28]. Nevertheless, multicollinearity is a common problem in logistic regression models where supposedly independent predictor variables are correlated. They tend to overestimate the variance of regression parameters and hinder the determination of the exact effect of each parameter, which could potentially result in inaccurate identification of significant predictors [29, 30]. In the paper, 115 HRnV parameters were derived but only seven variables were left in the final prediction model, and this implies the possible elimination of relevant information [28].

Within the general medical literature, machine learning dimensionality reduction methods are uncommon and limited to a few specific areas, such as bioinformatics studies on genetics [31, 32] and diagnostic radiological imaging [33, 34]. Despite this, dimensionality reduction in HRV has been investigated and shown to effectively compress multidimensional HRV data for the assessment of cardiac autonomic neuropathy [35]. In this paper, we attempted to investigate several machine learning dimensionality reduction algorithms in building predictive models, hypothesizing that these algorithms could be useful in preserving useful information while improving prediction performance. We aimed to compare the performance of the dimensionality reduction models against the traditional stepwise logistic regression model [28] and conventional risk stratification tools such as the HEART, TIMI, and GRACE scores, in the prediction of 30-day MACE in chest pain patients presenting to the ED.

## Methods

### Study design and clinical setting

A retrospective analysis was conducted on data collected from patients > 20 years old who presented to Singapore General Hospital ED with chest pain between September 2010 to July 2015. These patients were triaged using the Patient Acuity Category Scale (PACS) and those with PACS 1 or 2 were included in the study. Patients were excluded if they were lost to the 30-day follow-up or if they presented with ST-elevation myocardial infarction (STEMI) or non-cardiac etiology chest pain such as pneumothorax, pneumonia, and trauma as diagnosed by the ED physician. Patients with ECG findings that precluded quality HRnV analysis such as artifacts, ectopic beats, paced or non-sinus rhythm were also excluded.

### Data collection

For each patient, HRV and HRnV parameters were calculated using HRnV-Calc software suite [28, 36] from a five to six-minute single-lead (lead II) ECG performed via the X-series Monitor (ZOLL Medical, Corporation, Chelmsford, MA). Table 1 shows the full list of HRV and HRnV parameters used in this study. Besides, the first 12-lead ECGs taken during patients’ presentation to the ED were interpreted by two independent clinical reviewers and any pathological ST changes, T wave inversions, and Q-waves were noted. Patients’ demographics, medical history, first set of vital signs, and troponin-T values were obtained from the hospital’s electronic health records (EHR). In this study, high-sensitivity troponin-T was selected as the cardiac biomarker and an abnormal value was defined as > 0.03 ng/mL.

**Table 1:** List of traditional heart rate variability (HRV) and novel heart rate n-variability (HRnV) parameters used in this study. HRnV is a new representation of beat-to-beat variation in ECGs and parameter “n” controls the formation of new RR-intervals that are used for parameter calculation. Details of HRnV definition can be found in [28]

HRV	HR_**2**_V	HR_**2**_V_**1**_	HR_**3**_V	HR_**3**_V_**1**_	HR_**3**_V_**2**_
Mean NN	HR_2_V Mean NN	HR_2_V_1_ Mean NN	HR_3_V Mean NN	HR_3_V_1_ Mean NN	HR_3_V_2_ Mean NN
SDNN	HR_2_V SDNN	HR_2_V_1_ SDNN	HR_3_V SDNN	HR_3_V_1_ SDNN	HR_3_V_2_ SDNN
RMSSD	HR_2_V RMSSD	HR_2_V_1_ RMSSD	HR_3_V RMSSD	HR_3_V_1_ RMSSD	HR_3_V_2_ RMSSD
Skewness	HR_2_V Skewness	HR_2_V_1_ Skewness	HR_3_V Skewness	HR_3_V_1_ Skewness	HR_3_V_2_ Skewness
Kurtosis	HR_2_V Kurtosis	HR_2_V_1_ Kurtosis	HR_3_V Kurtosis	HR_3_V_1_ Kurtosis	HR_3_V_2_ Kurtosis
Triangular index	HR_2_V Triangular index	HR_2_V_1_ Triangular index	HR_3_V Triangular index	HR_3_V_1_ Triangular index	HR_3_V_2_ Triangular index
NN50	HR_2_V NN50	HR_2_V_1_ NN50	HR_3_V NN50	HR_3_V_1_ NN50	HR_3_V_2_ NN50
pNN50	HR_2_V pNN50	HR_2_V_1_ pNN50	HR_3_V pNN50	HR_3_V_1_ pNN50	HR_3_V_2_ pNN50
–	HR_2_V NN50*n*	HR_2_V_1_ NN50*n*	HR_3_V NN50*n*	HR_3_V_1_ NN50*n*	HR_3_V_2_ NN50*n*
–	HR_2_V pNN50*n*	HR_2_V_1_ pNN50*n*	HR_3_V pNN50*n*	HR_3_V_1_ pNN50*n*	HR_3_V_2_ pNN50*n*
Total power^a^	HR_2_V Total power	HR_2_V_1_ Total power	HR_3_V Total power	HR_3_V_1_ Total power	HR_3_V_2_ Total power
VLF power	HR_2_V VLF power	HR_2_V_1_ VLF power	HR_3_V VLF power	HR_3_V_1_ VLF power	HR_3_V_2_ VLF power
LF power	HR_2_V LF power	HR_2_V_1_ LF power	HR_3_V LF power	HR_3_V_1_ LF power	HR_3_V_2_ LF power
HF power	HR_2_V HF power	HR_2_V_1_ HF power	HR_3_V HF power	HR_3_V_1_ HF power	HR_3_V_2_ HF power
LF power norm	HR_2_V LF power norm	HR_2_V_1_ LF power norm	HR_3_V LF power norm	HR_3_V_1_ LF power norm	HR_3_V_2_ LF power norm
HF power norm	HR_2_V HF power norm	HR_2_V_1_ HF power norm	HR_3_V HF power norm	HR_3_V_1_ HF power norm	HR_3_V_2_ HF power norm
LF/HF	HR_2_V LF/HF	HR_2_V_1_ LF/HF	HR_3_V LF/HF	HR_3_V_1_ LF/HF	HR_3_V_2_ LF/HF
Poincaré SD1	HR_2_V Poincaré SD1	HR_2_V_1_ Poincaré SD1	HR_3_V Poincaré SD1	HR_3_V_1_ Poincaré SD1	HR_3_V_2_ Poincaré SD1
Poincaré SD2	HR_2_V Poincaré SD2	HR_2_V_1_ Poincaré SD2	HR_3_V Poincaré SD2	HR_3_V_1_ Poincaré SD2	HR_3_V_2_ Poincaré SD2
Poincaré SD1/SD2 ratio	HR_2_V Poincaré SD1/SD2	HR_2_V_1_ Poincaré SD1/SD2	HR_3_V Poincaré SD1/SD2	HR_3_V_1_ Poincaré SD1/SD2	HR_3_V_2_ Poincaré SD1/SD2
SampEn	HR_2_V SampEn	HR_2_V_1_ SampEn	HR_3_V SampEn	HR_3_V_1_ SampEn	HR_3_V_2_ SampEn
ApEn	HR_2_V ApEn	HR_2_V_1_ ApEn	HR_3_V ApEn	HR_3_V_1_ ApEn	HR_3_V_2_ ApEn
DFA, α1	HR_2_V DFA, α1	HR_2_V_1_ DFA, α1	HR_3_V DFA, α1	HR_3_V_1_ DFA, α1	HR_3_V_2_ DFA, α1
DFA, α2	HR_2_V DFA, α2	HR_2_V_1_ DFA, α2	HR_3_V DFA, α2	HR_3_V_1_ DFA, α2	HR_3_V_2_ DFA, α2

*Mean NN* average of R-R intervals, *SDNN* standard deviation of R-R intervals, *RMSSD* square root of the mean squared differences between R-R intervals, *NN50* the number of times that the absolute difference between 2 successive R-R intervals exceeds *50 ms* pNN50, NN50 divided by the total number of R-R intervals, *NN50n* the number of times that the absolute difference between 2 successive RR_*n*_I/RR_*n*_I_*m*_ sequences exceeds 50 × *n* ms, *pNN50n* NN50*n* divided by the total number of RR_*n*_I/RR_*n*_I_*m*_ sequences, *VLF* very low frequency, *LF* low frequency, *HF* high frequency, *SD* standard deviation, *SampEn* sample entropy, *ApEn* approximate entropy, *DFA* detrended fluctuation analysis

^a^In frequency domain analysis, the power of spectral components is the area below the relevant frequencies presented in absolute units (square milliseconds)

The primary outcome measured was any MACE within 30 days, including acute myocardial infarction, emergent revascularization procedures such as percutaneous coronary intervention (PCI) or coronary artery bypass graft (CABG), or death. The primary outcome was captured through a retrospective review of patients’ EHR.

### Machine learning dimensionality reduction

Dimensionality reduction in machine learning and data mining [37] refers to the process of transforming high-dimensional data into lower dimensions such that fewer features are selected or extracted while preserving essential information of the original data. Two types of dimensionality reduction approaches are available, namely variable selection and feature extraction. Variable selection methods generally reduce data dimensionality by choosing a subset of variables, while feature extraction methods transform the original feature space into lower-dimensional space through linear or nonlinear feature projection. In clinical predictive modeling, variable selection techniques such as stepwise logistic regression are popular for constructing prediction models [38]. In contrast, feature extraction approaches [39] are less commonly used in medical research, although they have been widely used in computational biology [40], image analysis [41, 42], physiological signal analysis [43], among others. In this study, we investigated the implementation of eight feature extraction algorithms and evaluated their contributions to prediction performance in risk stratification of ED chest pain patients. We also compared them with a prediction model that was built using conventional stepwise variable selection [28]. Henceforth, we use the terms “dimensionality reduction” and “feature extraction” interchangeably.

Given that there were *n* samples (***x***_*i*_, *y*_*i*_), *i* = 1, 2, …, *n*, in the dataset (*X*, *y*), where each sample *x*_*i*_ had original *D* features and its label *y*_*i*_ = 1 or 0, with 1 indicating a positive primary outcome, i.e., MACE within 30 days. We applied dimensionality reduction algorithms to project ***x***_*i*_ into a *d*-dimensional space (*d* < *D*). As a result, the original dataset ***X*** ∈ *ℝ*^*n* × *D*^ became $\hat{\boldsymbol{X}}\boldsymbol{\in}{\mathbb{R}}^{n\times d}$. There was a total of *D* = 174 candidate variables in this study. As suggested in Liu et al. [28], some variables were less statistically significant in terms of contributions to the prediction performance. Thus, we conducted univariable analysis and preselected a subset of $\overset{\sim }{D}$ variables if their $p<\overset{\sim }{P}$. In this study, we determined $\overset{\sim }{P}$ by running principal component analysis (PCA) [44] and logistic regression through 5-fold cross-validation; we plotted a curve to visualize the choice of a threshold and its impact on predictive performance. PCA was used for demonstration because of its simplicity and fast running speed. Other than PCA, we also implemented seven dimensionality reduction algorithms, including kernel PCA (KPCA) [45] with polynomial kernel function, latent semantic analysis (LSA) [46], Gaussian random projection (GRP) [47], sparse random projection (SRP) [48], multidimensional scaling (MDS) [49], Isomap [50], and locally linear embedding (LLE) [51]. All these algorithms are unsupervised learning methods, meaning the transformation of feature space does not rely on sample labels ***y***. Among the eight methods, MDS, Isomap, and LLE are manifold learning-based techniques for nonlinear dimensionality reduction. Table 2 gives a brief introduction to these eight methods.

**Table 2:** Summary of machine learning dimensionality reduction methods used in this study

Methods	Descriptions
Principal component analysis (PCA) [44]	PCA decomposes data into a set of successive orthogonal components that explain a maximum amount of the variance
Kernel PCA (KPCA) [45]	KPCA extends PCA by using kernel functions to achieve non-linear dimensionality reduction
Latent semantic analysis (LSA) [46]	LSA is similar to PCA but differs in that the data matrix does not need to be centered
Gaussian random projection (GRP) [47]	GRP projects the original input features onto a randomly generated matrix where components are drawn from a Gaussian distribution
Sparse random projection (SRP) [48]	SRP projects the original input features onto a sparse random matrix, which is an alternative to dense Gaussian random projection matrix
Multidimensional scaling (MDS) [49]	MDS is a technique used for analyzing similarity or dissimilarity data, seeking a low-dimensional representation of the data in which the distances respect well the distances in the original high-dimensional space
Isomap [50]	Isomap is a manifold learning algorithm, seeking a lower-dimensional embedding that maintains geodesic distances between all points
Locally linear embedding (LLE) [51]	LLE projects the original input features to a lower-dimensional space by preserving distances within local neighborhoods

### Predictive and statistical analysis

In this study, we chose logistic regression as the classification algorithm to predict the MACE outcome. As described earlier, we determined the threshold $\overset{\sim }{P}$ to preselect a subset of $\overset{\sim }{D}$ variables, ensuring the removal of less significant variables as indicated by univariable analysis, after which *X* ∈ *ℝ*^*n* × *D*^ became $\overset{\sim }{X}\in {\mathbb{R}}^{n\times \overset{\sim }{D}}$. In summary, the inputs to all dimensionality reduction algorithms were in $\overset{\sim }{D}$-dimensional space. Subsequently, conventional logistic regression was implemented to take *d*-dimensional $\hat{\boldsymbol{X}}$ to predict ***y***, where 5-fold cross-validation was used.

We compared the models built with machine learning dimensionality reduction with our previous stepwise model [28], in which the following 16 variables were used: age, diastolic blood pressure, pain score, ST-elevation, ST-depression, Q wave, cardiac history (the “History” component in the HEART score), troponin, HRV NN50, HR_2_V skewness, HR_2_V SampEn, HR_2_V ApEn, HR_2_V_1_ ApEn, HR_3_V RMSSD, HR_3_V skewness, and HR_3_V_2_ HF power. As described in [28], we selected candidate variables with *p* < 0.2 in univariable analysis and subsequently conducted multivariable analysis using backward stepwise logistic regression. In the current study, we further built eight dimensionality reduction models without using the cardiac troponin and compared them with the stepwise model without the troponin component. This analysis enabled us to check the feasibility of avoiding the use of laboratory results for quick risk stratification.

In evaluating the modeling performance, we performed the receiver operating characteristic (ROC) curve analysis and reported the corresponding area under the curve (AUC), sensitivity, specificity, positive predictive value (PPV), and negative predictive value (NPV) measures. Moreover, we generated the calibration plots for prediction models. In describing the data, we reported continuous variables as the median and interquartile range (IQR) and statistical significance using two-sample t-test. We reported categorical variables as frequency and percentage and statistical significance using chi-square test. All analyses were conducted in Python version 3.8.0 (Python Software Foundation, Delaware, USA).

## Results

We included 795 chest pain patients in this study, of which 247 (31%) patients had MACE within 30 days of presentation to the ED. Table 3 presents the baseline characteristics of the patient cohort. Patients with MACE were older (median age 61 years vs. 59 years, *p* = 0.035) and more likely to be male (76.1% vs. 64.6%, *p* = 0.002). History of diabetes, current smoking status, and pathological ECG changes such as ST elevation, ST depression, T wave inversion, pathological Q waves, and QTc prolongation were significantly more prevalent in patients with the primary outcome. Troponin-T and creatine kinase-MB levels were also significantly elevated in patients with the primary outcome. There was no statistically significant difference in patient ethnicity between MACE and non-MACE groups.

**Table 3:** Baseline characteristics of patient cohorts

	Total (***n*** = 795)	MACE (***n*** = 247)	Non-MACE (***n*** = 548)	***p***-value
**Age, median (IQR)**	60 (51–68)	61 (54–68)	59 (50–68)	0.035
**Male gender, n (%)**	542 (68.2)	188 (76.1)	354 (64.6)	0.002
**Race, n (%)**				0.623
Chinese	492 (61.9)	159 (64.4)	333 (60.8)	0.374
Indian	129 (16.2)	34 (13.8)	95 (17.3)	0.246
Malay	150 (18.9)	46 (18.6)	104 (19.0)	0.984
Other	24 (3.0)	8 (3.2)	16 (2.9)	0.984
**Vital signs, median (IQR)**				
Temperature (°C)	36.4 (36.0–36.7)	36.3 (36.0–36.7)	36.4 (36.0–36.7)	0.793
Heart rate (beats/min)	76 (67–89)	80 (69–92.5)	75 (66–87)	0.03
Respiratory rate (breaths/min)	18 (17–18)	18 (17–18)	18 (17–18)	0.716
Systolic blood pressure (mmHg)	138 (123.0–159.0)	142 (123.5–165.5)	137 (122.0–156.2)	0.037
Diastolic blood pressure (mmHg)	76.0 (68.0–86.0)	78.0 (70.0–89.0)	75.0 (67.0–84.0)	0.001
SpO_2_ (%)	99.0 (97.0–100.0)	99.0 (97.0–100.0)	99.0 (97.0–100.0)	0.842
Pain score	2.0 (0.0–5.0)	2.0 (0.0–5.0)	2.0 (0.0–5.0)	0.008
Glasgow Coma Scale (GCS) score	15.0 (15.0–15.0)	15.0 (15.0–15.0)	15.0 (15.0–15.0)	0.121
**Medical history, n (%)**				
Ischaemic heart disease	343 (43.1)	115 (46.6)	228 (41.6)	0.22
Diabetes	278 (35.0)	106 (42.9)	172 (31.4)	0.002
Hypertension	509 (64.0)	161 (65.2)	348 (63.5)	0.707
Hypercholesterolaemia	476 (59.9)	151 (61.1)	325 (59.3)	0.683
Stroke	58 (7.3)	15 (6.1)	43 (7.8)	0.458
Cancer	29 (3.6)	7 (2.8)	22 (4.0)	0.537
Respiratory disease	31 (3.9)	5 (2.0)	26 (4.7)	0.102
Chronic kidney disease	87 (10.9)	26 (10.5)	61 (11.1)	0.896
Congestive heart failure	38 (4.8)	9 (3.6)	29 (5.3)	0.407
History of PCI	199 (25.0)	68 (27.5)	131 (23.9)	0.316
History of CABG	71 (8.9)	26 (10.5)	45 (8.2)	0.355
History of AMI	133 (16.7)	48 (19.4)	85 (15.5)	0.205
Active smoker	197 (24.8)	73 (29.6)	124 (22.6)	0.045
**ECG pathology, n (%)**				
ST elevation	65 (8.2)	48 (19.4)	17 (3.1)	< 0.001
ST depression	92 (11.6)	69 (27.9)	23 (4.2)	< 0.001
T wave inversion	209 (26.3)	86 (34.8)	123 (22.4)	< 0.001
Pathological Q wave	86 (10.8)	51 (20.6)	35 (6.4)	< 0.001
QTc prolongation	174 (21.9)	73 (29.6)	101 (18.4)	0.001
Left axis deviation	64 (8.1)	16 (6.5)	48 (8.8)	0.34
Right axis deviation	16 (2.0)	6 (2.4)	10 (1.8)	0.773
Left bundle branch block	8 (1.0)	3 (1.2)	5 (0.9)	0.991
Right bundle branch block	56 (7.0)	14 (5.7)	42 (7.7)	0.385
Interventricular conduction delay	30 (3.8)	13 (5.3)	17 (3.1)	0.201
Left atrial abnormality	12 (1.5)	4 (1.6)	8 (1.5)	0.886
Left ventricular hypertrophy	103 (13.0)	38 (15.4)	65 (11.9)	0.21
Right ventricular hypertrophy	6 (0.8)	1 (0.4)	5 (0.9)	0.747
**Laboratory findings, median (IQR)**				
Troponin (ng/L)	0 (0–39.5)	40 (10–170)	0 (0–15.2)	< 0.001
Creatine kinase-MB	2.4 (1.8–3.2)	2.7 (2.1–6.0)	2.4 (1.7–2.7)	< 0.001
**Clinical scores, median (IQR)**				
HEART	5.0 (4.0 to 7.0)	7.0 (6.0 to 8.0)	4.0 (3.0 to 6.0)	< 0.001
TIMI	2.0 (1.0 to 4.0)	3.0 (2.0 to 4.0)	2.0 (1.0 to 3.0)	< 0.001
GRACE	104.0 (83.5 to 128.0)	119.0 (97.0 to 139.0)	98.0 (78.0 to 125.0)	< 0.001

*IQR* interquartile range, *MACE* major adverse cardiac events, *PCI* percutaneous coronary intervention, *CABG* coronary artery bypass graft, *AMI* acute myocardial infarction, *HEART* History, ECG, Age, Risk factors and Troponin, *TIMI* Thrombolysis in Myocardial Infarction, *GRACE* Global Registry of Acute Coronary Events

**Fig. 1:**
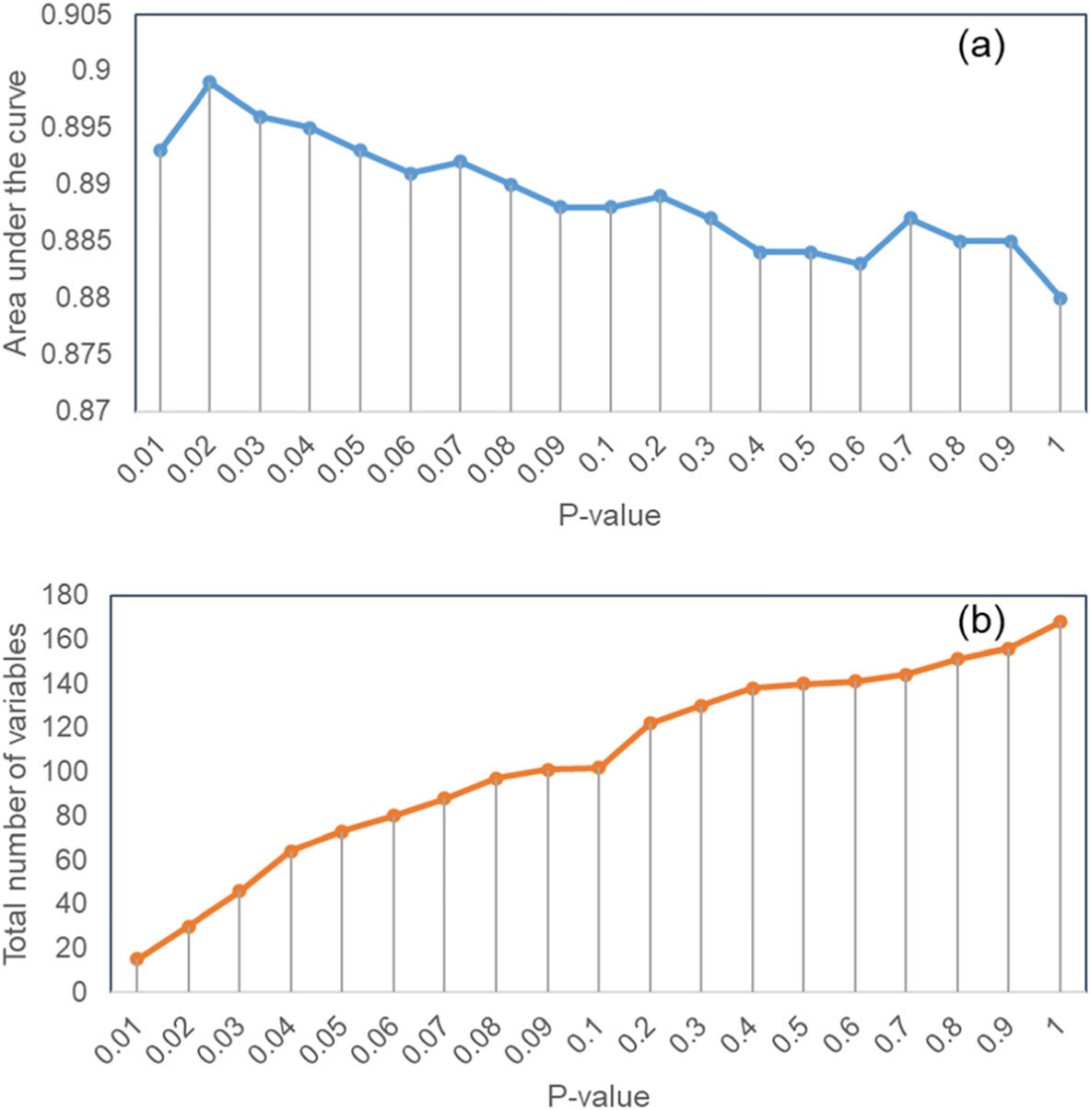
Variable preselection using *p*-value in univariable analysis for dimensionality reduction: **a** prediction area under the curve versus the *p*-value, and (**b**) the number of preselected variables versus the *p*-value

Figure 1a depicts the PCA-based predictive performance versus the threshold $\overset{\sim }{P}$ (for preselection of variables) and Fig. 1b shows the number of preselected variables versus threshold $\overset{\sim }{P}$. The predictive performance peaked at $\overset{\sim }{P}=0.02$, where a total of 30 variables were preselected, including gender, diastolic blood pressure, pain score, ST-elevation, ST-depression, T-wave inversion, Q wave, cardiac history, EKG, and risk factor components of the HEART score, troponin, HRV RMSSD, HRV NN50, HRV pNN50, HRV HF power, HRV Poincaré SD1, HR_2_V RMSSD, HR_2_V NN50, HR_2_V pNN50, HR_2_V HF power, HR_2_V Poincaré SD1, HR_2_V_1_ RMSSD, HR_2_V_1_ NN50, HR_2_V_1_ HF power, HR_2_V_1_ Poincaré SD1, HR_3_V_1_ RMSSD, HR_3_V_1_ HF power, HR_3_V_1_ Poincaré SD1, HR_3_V_2_ RMSSD, and HR_3_V_2_ Poincaré SD1. These were used as inputs to all dimensionality reduction algorithms whose outputs were linear or nonlinear combinations of these 30 variables.

**Fig. 2:**
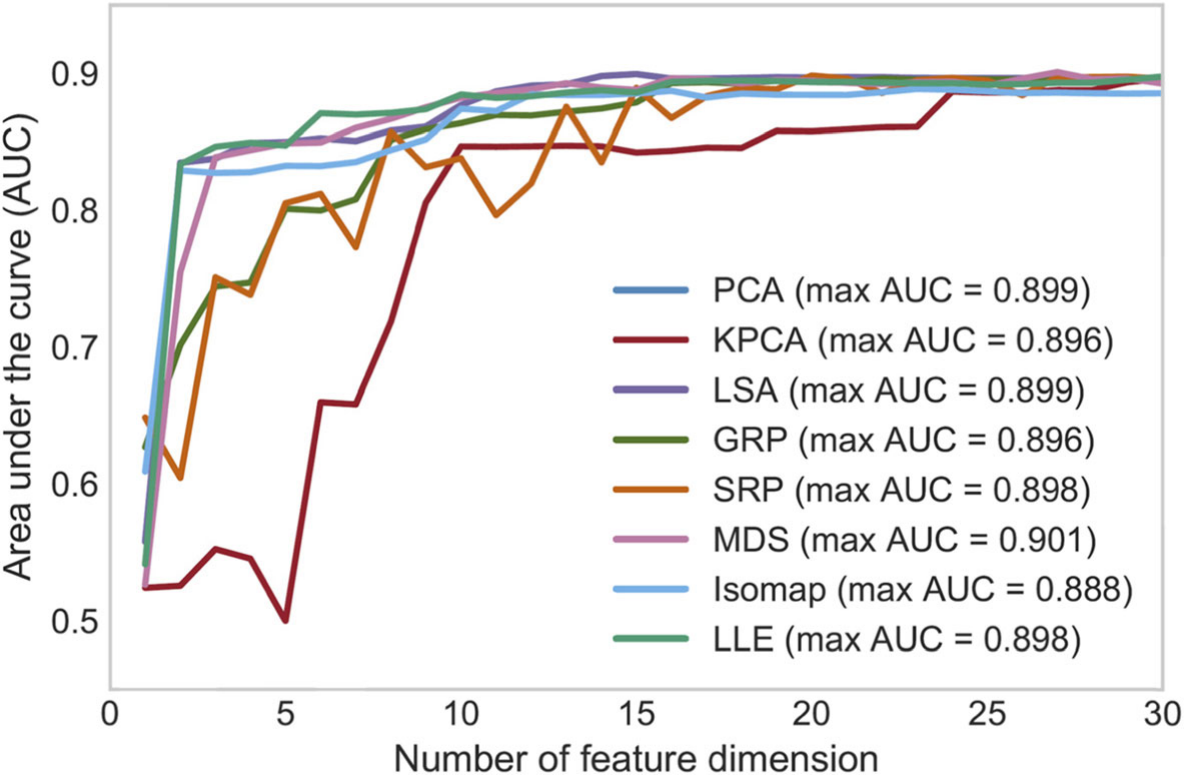
Prediction performance based on the eight dimensionality reduction algorithms versus the number of feature dimensions after reduction

Figure 2 shows the predictive performance (in terms of AUC value) versus feature dimension (i.e., number of “principal components”) for all eight dimensionality reduction algorithms. The AUC values of GRP, SRP, and KPCA gradually increased with the increment of feature dimension, while the AUC values of PCA, LSA, MDS, Isomap, and LLE drastically jumped to more than 0.8 when feature dimension *d* ≥ 3 and plateaued in the curves when *d* ≥ 15. The highest AUC values of PCA, KPCA, LSA, GRP, SRP, MDS, Isomap, and LLE were 0.899, 0.896, 0.899, 0.896, 0.898, 0.901, 0.888, and 0.898, achieved with feature dimensions of 15, 30, 15, 22, 20, 27, 23, and 30, respectively.

**Fig. 3:**
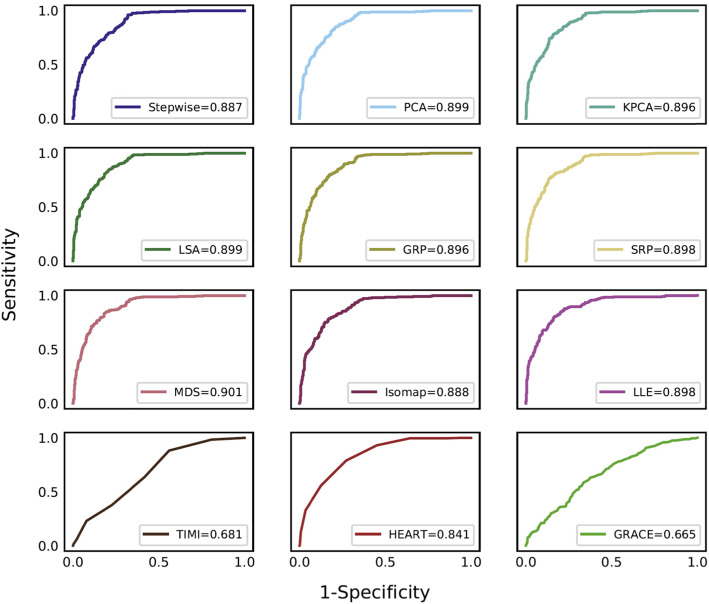
ROC curves (based on the optimal number of dimensions) generated by the stepwise model, eight dimensionality reduction models, and three clinical scores

**Fig. 4:**
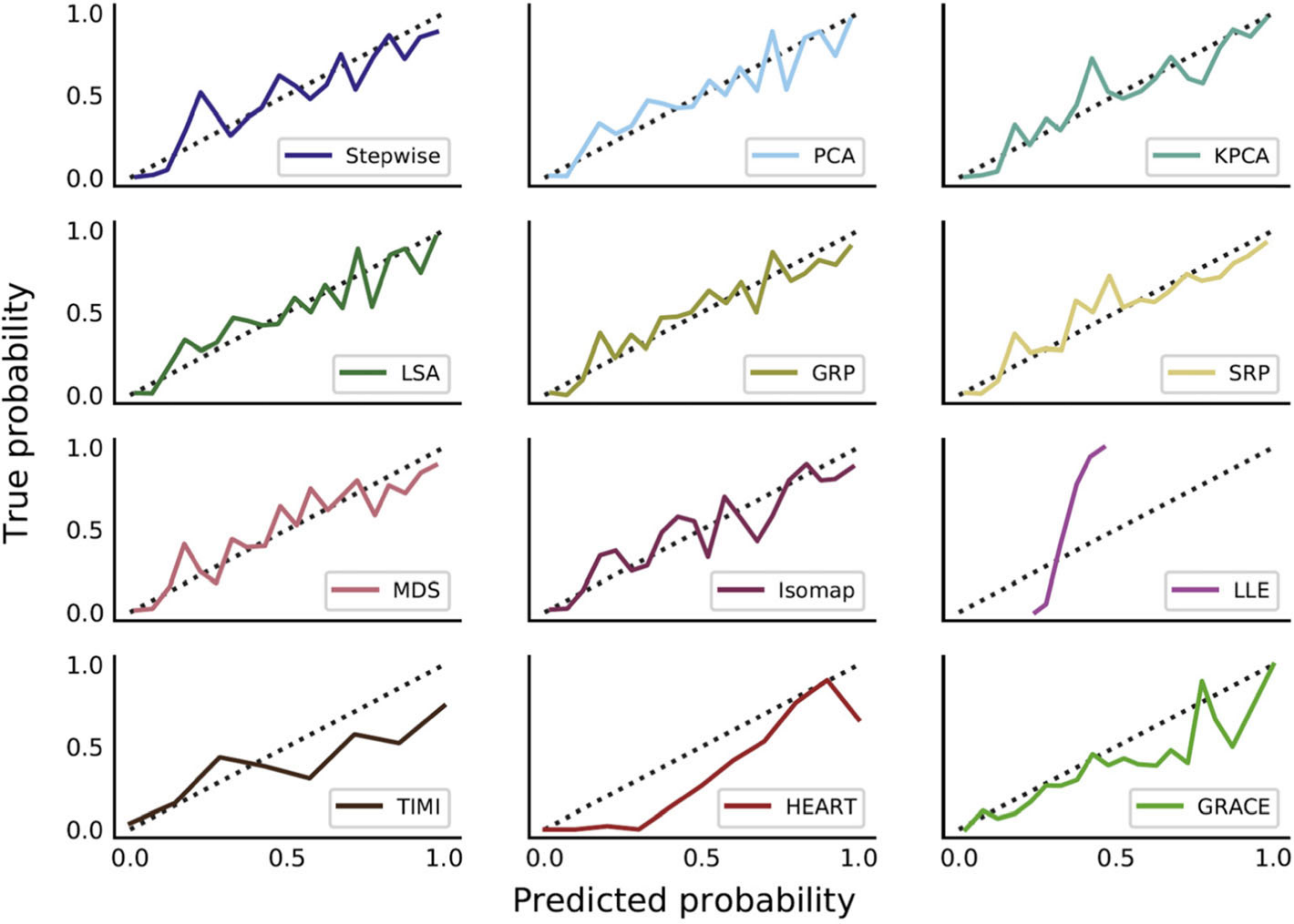
Calibration curves (based on the optimal number of dimensions) generated by the stepwise model, eight dimensionality reduction models, and three clinical scores

Figure 3 shows the ROC curves of the eight dimensionality reduction algorithms, the stepwise logistic regression [28], and three clinical scores. All eight dimensionality reduction methods performed comparably with the stepwise variable selection, and MDS achieved the highest AUC of 0.901. Table 4 presents ROC analysis results of all 12 methods/scores where sensitivity, specificity, PPV, and NPV are reported with 95% confidence intervals (CIs), noting that the performance of the stepwise model in this paper was slightly different from that reported in [28] due to the choice of cross-validation scheme, i.e., 5-fold (AUC of 0.887) versus leave-one-out (AUC of 0.888). Figure 4 presents the calibration curves of predictions by all methods/scores. The stepwise model and seven dimensionality reduction models (PCA, KPCA, LSA, GRP, SRP, MDS, and Isomap) showed reasonable model calibrations, in which their curves fluctuated along the diagonal line, meaning these models only slightly overestimated or underestimated the predicted probability of 30-day MACE. The LLE model was unable to achieve good calibration. In comparison, all three clinical scores (HEART, TIMI, and GRACE) generally underpredicted the probability of 30-day MACE.

**Table 4:** Comparison of performance of the HRnV models (based on 5-fold cross-validation), HEART, TIMI, and GRACE scores in predicting 30-day major adverse cardiac events (MACE). The cut-off values were defined as the points nearest to the upper-left corner on the ROC curves

	AUC (95% CI)	Cut-off	Sensitivity % (95% CI)	Specificity % (95% CI)	PPV % (95% CI)	NPV % (95% CI)
**Stepwise**	0.887 (0.859–0.916)	0.3140	79.4 (74.3–84.4)	78.8 (75.4–82.3)	62.8 (57.5–68.2)	89.4 (86.7–92.2)
**PCA**	0.899 (0.872–0.926)	0.2881	85.4 (81.0–89.8)	78.5 (75.0–81.9)	64.1 (59.0–69.3)	92.3 (89.9–94.7)
**KPCA**	0.896 (0.869–0.923)	0.3489	81.8 (77.0–86.6)	82.1 (78.9–85.3)	67.3 (62.0–72.6)	90.9 (88.4–93.4)
**LSA**	0.899 (0.872–0.926)	0.2884	85.4 (81.0–89.8)	78.6 (75.2–82.1)	64.3 (59.1–69.5)	92.3 (89.9–94.7)
**GRP**	0.896 (0.868–0.923)	0.2965	85.0 (80.6–89.5)	78.5 (75.0–81.9)	64.0 (58.8–69.2)	92.1 (89.6–94.5)
**SRP**	0.898 (0.871–0.925)	0.2940	84.6 (80.1–89.1)	79.6 (76.2–82.9)	65.1 (59.9–70.3)	92.0 (89.5–94.4)
**MDS**	0.901 (0.874–0.928)	0.3095	83.4 (78.8–88.0)	81.6 (78.3–84.8)	67.1 (61.8–72.4)	91.6 (89.1–94.1)
**Isomap**	0.888 (0.860–0.917)	0.3468	78.5 (73.4–83.7)	82.7 (79.5–85.8)	67.1 (61.7–72.5)	89.5 (86.9–92.2)
**LLE**	0.898 (0.870–0.925)	0.3140	85.0 (80.6–89.5)	79.4 (76.0–82.8)	65.0 (59.8–70.2)	92.2 (89.7–94.6)
**HEART**	0.841 (0.808–0.874)	5	78.9 (73.9–84.0)	72.8 (69.1–76.5)	56.7 (51.4–61.9)	88.5 (85.5–91.4)
**TIMI**	0.681 (0.639–0.723)	2	63.6 (57.6–69.6)	58.4 (54.3–62.5)	40.8 (35.9–45.7)	78.0 (74.0–82.1)
**GRACE**	0.665 (0.623–0.707)	107	64.0 (58.0–70.0)	60.8 (56.7–64.9)	42.4 (37.3–47.4)	78.9 (75.0–82.8)

*AUC* area under the curve, *CI* confidence interval, *PPV* positive predictive value, *NPV* negative predictive value, *HEART* History, ECG, Age, Risk factors and Troponin, *TIMI* Thrombolysis in Myocardial Infarction, *GRACE* Global Registry of Acute Coronary Events

**Fig. 5:**
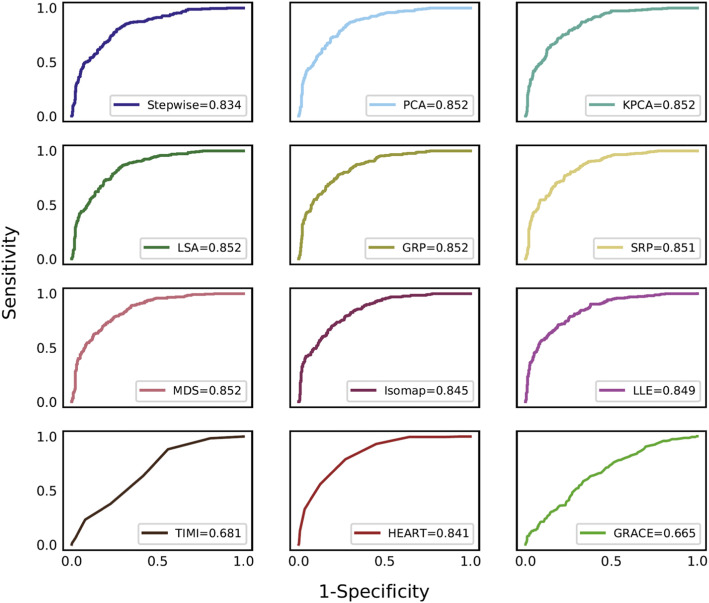
ROC curves (based on the optimal number of dimensions) generated by the stepwise model, eight dimensionality reduction models, and three clinical scores, where the prediction models were built without using cardiac troponin.s

Figure 5 shows the ROC curves of prediction models without using cardiac troponin. At feature dimensions of 13, 21, 13, 29, 24, 17, 18, and 18, the highest AUC values of PCA, KPCA, LSA, GRP, SRP, MDS, Isomap, and LLE were 0.852, 0.852, 0.852, 0.852, 0.851, 0.852, 0.845, and 0.849, respectively. The stepwise model without troponin yielded an AUC of 0.834 compared to 0.887 with troponin. All prediction models outperformed both the TIMI and GRACE scores while achieving comparable results with the HEART score.

## Discussion

In this study, we showed that machine learning dimensionality reduction yielded only marginal, non-significant improvements compared to stepwise model in predicting the risk of 30-day MACE among chest pain patients in the ED. This corroborates with similar observations that traditional statistical methods can perform comparably to machine learning algorithms [52, 53]. Among the dimensionality reduction models integrated with cardiac troponin, the MDS model had the highest discriminative performance (AUC of 0.901, 95% CI 0.874–0.928) but did not significantly outperformed the traditional stepwise model (AUC of 0.887, 95% CI 0.859–0.916). Among the models without using troponin, PCA, KPCA, LSA, GRP, and MDS performed equally well, achieving an AUC of 0.852, compared with the stepwise model without troponin which had an AUC of 0.834. In general, the traditional stepwise approach was proved to be comparable to machine learning dimensionality reduction methods in risk prediction, while benefiting from model simplicity, transparency, and interpretability that are desired in real-world clinical practice.

High-dimensional data suffers from the curse of dimensionality, which refers to the exponentially increasing sparsity of data and sample size required to estimate a function to a given accuracy as dimensionality increases [54]. Dimensionality reduction has successfully mitigated the curse of dimensionality in the analysis of high-dimensional data in various domains such as computational biology and bioinformatics [31, 32]. However, clinical predictive modeling typically considers relatively few features, limiting the effects of the curse of dimensionality. This may account for the relatively limited benefit of dimensionality reduction in our analysis.

Additionally, with comparable performance to the traditional stepwise model, transparency and interpretability of machine learning dimensionality reduction models are constrained by complex algorithmic transformations of variables, leading to obstacles in the adoption of such models in real-world clinical settings. In contrast, traditional biostatistical approaches like logistic regression with stepwise variable selection deliver a simple and transparent model, in which the absolute and relative importance of each variable can be easily interpreted and explained from the odds ratio. Marginal performance improvements should be weighed against these limitations in interpretability, which is an important consideration in clinical predictive modeling.

Comparing the eight dimensionality reduction algorithms, PCA and LSA use common linear algebra techniques to learn to create principal components in a compressed data space, while MDS, Isomap, and LLE are nonlinear, manifold learning-based dimensionality reduction methods. As observed from our results, complex nonlinear algorithms did not show an obvious advantage over simple PCA and LSA methods in enhancing the predictive performance. Yet, nonlinear algorithms are more computationally complex and require more computing memory. For example, KPCA and Isomap have computational complexity of *O*(*n*^3^) and memory complexity of *O*(*n*^2^), while PCA has computational complexity of $O\left({\overset{\sim }{D}}^3\right)$ and memory complexity of $O\left({\overset{\sim }{D}}^2\right)$ [39]. In applications of clinical predictive modeling, *n* ⁠— the number of patients ⁠— is usually larger than $\overset{\sim }{D}$ ⁠ — the number of variables; in our study, *n* is 795 and $\overset{\sim }{D}$ is 29 or 30, depending on the inclusion of troponin. This suggests that linear algorithms may be preferred due to reduced computational complexity and memory while retaining comparable performance. Another observation in this study was that the impact of preselection (as shown in Fig. 1) on predictive performance was more substantial than that of dimensionality reduction, indicating the importance of choosing statistically significant candidate variables.

Our study also reiterates the value of HRnV-based prediction models for chest pain risk stratification. Among chest pain risk stratification tools in the ED, clinical scores like HEART, TIMI, and GRACE are currently the most widely adopted and validated [55, 56]. However, a common barrier to quick risk prediction using these traditional clinical scores is the requirement of cardiac troponin, which can take hours to obtain. To address these difficulties, machine learning-based predictive models that integrate HRV measures and clinical parameters have been proposed [17, 22, 25, 26], including our development of HRnV, a novel alternative measure to HRV that has shown promising results in predicting 30-day MACE [28], which was the stepwise model in this paper. Both the dimensionality reduction-based predictive models and the stepwise model with troponin presented superior performance than HEART, TIMI, and GRACE scores. When troponin was not used, several dimensionality reduction-based models such as PCA, KPCA, and MDS still yielded marginally better performance than the original HEART score, while benefiting from generating the predictive scores in merely 5 to 6 min.

Additionally, Table 4 shows that all HRnV-based predictive models had higher specificities than the HEART score while all HRnV-based models except Isomap also improved on the already high sensitivity of the HEART score [21, 57]. The specificities of KPCA, Isomap, and MDS were significantly higher by an absolute value of almost 10%. Substantial improvements to the specificity of MACE predictive models may reduce unnecessary admission and thus minimize costs and resource usage [5]. This is particularly relevant in low-resource settings, for example, the overburdened EDs in the current coronavirus disease 2019 (COVID-19) pandemic, where novel methods in resource allocation and risk stratification could alleviate the strain on healthcare resources [58].

There remains a need for further investigation into methods that utilize information from the full set of HRV and HRnV variables. From 174 variables in the initial data set, dimensionality reduction performed the best with a preselection of 30 variables, of which 19 were HRV and HRnV parameters. That is, the majority of the newly constructed HRnV parameters were removed based on the strict significance threshold of *p* < 0.02 on univariable analysis. Therefore, novel HRnV measures were not fully used in prediction models of 30-day MACE, leaving room for further investigation of alternative ways of using them. Moving forward, it may be valuable to develop and evaluate deep learning frameworks [59] to synthesize novel low-dimensional representations of multidimensional information. Alternatively, building point-based, interpretable risk scores [60] can also be beneficial to implementation and adoption in real-world clinical settings, since designing inherently interpretable models is more favorable than explaining black box models [61].

We acknowledge the following limitations of this study. First, the clinical application (i.e., risk stratification of ED chest pain patients) was only one example of clinical predictive modeling, thus our conclusion on the effectiveness of machine learning dimensionality reduction algorithms may not be generalizable to other applications, particularly those with a larger number of variables. Second, only eight dimensionality reduction algorithms were investigated, while many other methods are available. Third, given the small sample size, we were unable to determine the threshold $\overset{\sim }{P}$ and build predictive models with a separate training set; this also limited the stability check [62] for both logistic regression and machine learning models. Last, we did not build a workable predictive model for risk stratification of ED chest pain patients, although several models built in this study showed promising results compared to existing clinical scores. We aim to conduct further investigations.

## Conclusions

In this study we found that machine learning dimensionality reduction models showed marginal value in improving the prediction of 30-day MACE for ED chest pain patients. Being black box models, they are further constrained in clinical practice due to low interpretability. Whereas traditional stepwise prediction model showed simplicity and transparency, making it feasible for clinical use. To fully utilize the available information in building high-performing predictive models, we suggest additional investigations such as exploring deep representations of the input variables and creating interpretable machine learning models to facilitate real-world clinical implementation.

## Data Availability

The datasets used and/or analyzed during the current study are available from the corresponding author on reasonable request.

## Abbreviations

ACS: Acute coronary syndrome
AMI: Acute myocardial infarction
AUC: Area under the curve
ApEn: Approximate entropy
CI: Confidence intervals
CABG: Coronary artery bypass graft
COVID-19: Coronavirus disease 2019
DFA: Detrended fluctuation analysis
ECG: Electrocardiogram
EHR: Electronic health records
ED: Emergency department
GRP: Gaussian random projection
GRACE: Global registry of acute coronary events
HRnV: Heart rate n-variability
HRV: Heart rate variability
HF: High frequency
HEART: History, ECG, Age, Risk factors, and initial Troponin
IQR: Interquartile range
KPCA: Kernel principal component analysis
LSA: Latent semantic analysis
LLE: Locally linear embedding
LF: Low frequency
MACE: Major adverse cardiac events
Mean NN: Average of R-R intervals
MDS: Multidimensional scaling
NPV: Negative predictive value
NN50: The number of times that the absolute difference between 2 successive R-R intervals exceeds 50 ms
NN50*n*: The number of times that the absolute difference between 2 successive RR_*n*_I/RR_*n*_I_*m*_ sequences exceeds 50 × *n* ms
PACS: Patient acuity category scale
PCI: Percutaneous coronary intervention
pNN50: NN50 divided by the total number of R-R intervals
pNN50*n*: NN50*n* divided by the total number of RR_*n*_I/RR_*n*_I_*m*_ sequences
PPV: Positive predictive value
PCA: Principal component analysis
ROC: Receiver operating characteristic
RMSSD: Square root of the mean squared differences between R-R intervals
RRI: R-R interval
SampEn: Sample entropy
SD: Standard deviation
SDNN: Standard deviation of R-R intervals
SRP: Sparse random projection
STEMI: ST-elevation myocardial infarction
TIMI: Thrombolysis in myocardial infarction
VLF: Very low frequency
